# Falls in Post-Polio Patients: Prevalence and Risk Factors

**DOI:** 10.3390/biology10111110

**Published:** 2021-10-28

**Authors:** Yonah Ofran, Isabella Schwartz, Sheer Shabat, Martin Seyres, Naama Karniel, Sigal Portnoy

**Affiliations:** 1 Faculty of Medicine, Hebrew University of Jerusalem, Jerusalem 91905, Israel; yonaho@hadassah.org.il (Y.O.); isabellas@hadassah.org.il (I.S.); sheersha@hadassah.org.il (S.S.); martin.seyres@mail.huji.ac.il (M.S.); 2 Department of Physical Medicine & Rehabilitation, Hadassah University Hospital, Jerusalem 9765418, Israel; dkar435@hadassah.org.il; 3 Department of Occupational Therapy, Sackler Faculty of Medicine, Tel Aviv University, Tel Aviv 6997801, Israel

**Keywords:** gait analysis, coefficient of variability, gait symmetry, timed up and go

## Abstract

**Simple Summary:**

People with post-polio syndrome (PPS) suffer frequent falls due to muscle weakness and problems with their balance. In order for a rehabilitation clinician to fit the patient with the optimal treatment plan to prevent imbalance and falls, we performed a simple 10-min walking test with 50 PPS patients. We also asked the patients how many falls they had experienced in the last year and they filled out a questionnaire regarding their balance confidence. We found that we can predict the occurrence of falls in PPS patients based on the consistency of their walking pattern. Since it is very easy to measure the walking pattern, our results may help rehabilitation clinicians to identify individuals at risk of fall and reduce the occurrence of falls in this population.

**Abstract:**

Individuals with post-polio syndrome (PPS) suffer from falls and secondary damage. Aim: To (i) analyze the correlation between spatio-temporal gait data and fall measures (fear and frequency of falls) and to (ii) test whether the gait parameters are predictors of fall measures in PPS patients. Methods: Spatio-temporal gait data of 50 individuals with PPS (25 males; age 65.9 ± 8.0) were acquired during gait and while performing the Timed Up-and-Go test. Subjects filled the Activities-specific Balance Confidence Scale (ABC Scale) and reported number of falls during the past year. Results: ABC scores and number of falls correlated with the Timed Up-and-Go, and gait cadence and velocity. The number of falls also correlated with the swing duration symmetry index and the step length variability. Four gait variability parameters explained 33.2% of the variance of the report of falls (*p* = 0.006). The gait velocity was the best predictor of the ABC score and explained 24.8% of its variance (*p* = 0.001). Conclusion: Gait variability, easily measured by wearables or pressure-sensing mats, is an important predictor of falls in PPS population. Therefore, gait variability might be an efficient tool before devising a patient-specific fall prevention program for the PPS patient.

## 1. Introduction

Fall frequency among individuals with post-polio syndrome (PPS) is estimated at approximately 70%, and one-third of those who fall sustain fragility fractures in the polio-involved limb [1,2]. Fall management programs for the PPS population mostly aim to manage asymmetric gait and reduce energy requirements [3]. Gait is mainly improved by assistive devices [4] and orthoses, balance training, and knee extensor strengthening exercises. 

Non-fatiguing strengthening and exercises involving isokinetic, isometric, and endurance muscular training, have been demonstrated to improve symptoms of muscular fatigue and pain in patients with PPS [5,6,7]. Reduced energy requirements are advocated to diminish the symptoms of fatigue. This is obtained by pacing strategies, including scheduling rest periods during the day, as well as during activities, and general lifestyle modifications, including weight control and modification of daily activities [8].

One of the factors that contributes to the occurrence of falls in PPS patients is extensive muscle weakness, particularly of the knee extensors of the affected leg. This weakness exists either as a remnant of the primary infection or appears and progresses later in life as a part of PPS. Another risk factor for falls is the fear of falling. Previous studies reported fear of falling in 60–95% of PPS patients [1,9,10,11]. Additional risk factors for falls in PPS patients include fatigue, muscle and joint pain, reduced sensation in the legs, depression, reduced gait performance, and impaired dynamic balance [1,11,12,13].

Imbalance in PPS patients can be explained by asymmetric gait, caused by asymmetrical involvement of the muscles, chronic compensatory, musculoskeletal deformities or contractures, and excessive surgical or orthotic alternations. Balance confidence in this population might also be impaired by reduced proprioceptive input during muscle contraction, caused by altered sensibility of muscle spindles at different contraction levels [14].

Post-polio syndrome patients may develop chronic compensatory musculoskeletal deformities or contractures, which allows them to increase ambulation speed and balance. However, excessive alternation of these compensatory mechanisms by an orthosis may worsen the mobility of the patient and induce falls [15]. A consequence of the deformities can be quantified by leg-length discrepancy, which was found to be another predictor of falls in PPS patients [2].

Presently, the acquirement of spatio-temporal gait data—e.g., step length and stance duration—has become frequent, due to the availability of simple wearable hardware or pressure-sensing mats (see Table 1 summarizing previous studies of PPS gait). These data hold within them variables of symmetry and variation, which provide important characterization of the PPS gait. However, while these data have been thoroughly quantified for PPS patients [16], their ability to predict falls, has yet to be explored. As summarized in Table 1, only four of the reviewed studies that performed instrumented gait analysis with PPS patients, collected data relating to fall or fear of fall. Two of which used low-technology (stop watch and pedometer) so that calculation of gait symmetry or variability could not be achieved. The remaining two researchers did not use the data to find which gait parameters best predict falls in this population. We therefore aimed (i) to analyze the correlation between spatio-temporal gait data and fall measures (fear and frequency of falls); and (ii) to test whether the gait parameters are predictors of the aforementioned fall measures in PPS patients. 

## 2. Methods and Materials

### 2.1. Population

We recruited 50 patients with PPS (25 males; average and SD age of 65.9 ± 8.0 years). Inclusion criteria were: PPS patients age 18 to 80, at least 15 years following the acute illness, independent walking ability for at least 10 m (with or without walking aids). Exclusion criteria: degenerative neurological disability that is not secondary to the PPS, unstable state of health (e.g., heart disease, respiratory insufficiency, peripheral vascular disease, acquired brain injury), inability to persevere and cooperate in the series of tests. Seventeen had weakness on their left side, 27 on their right side, and 6 on both sides. The average and SD of leg length discrepancy was 3.1 ± 1.6 cm. Twenty-two did not use walking aids, 4 used a crutch, 8 used two crutches, 11 used a cane, 4 used a walker, and 1 used Nordic sticks. Additionally, 23 of the subjects used orthoses (13 used a knee-ankle-foot orthoses, 5 used an ankle-foot orthosis, and 4 used a Dictus). Twenty-seven subjects used orthopedic shoes and 12 occasionally used a wheelchair.

Ethical approval was granted by the Hadassah medical center Helsinki committee pretrial (approval number HMO-0162-19). All of the subjects read and signed an informed consent form.

### 2.2. Tools and Protocol

The following questionnaires were filled by each subject: demographic questionnaire, The Activities-specific Balance Confidence Scale (ABC Scale) [27] which is a subjective 16-item measure of confidence in performing various ambulatory activities without falling or experiencing a sense of unsteadiness. Items are rated between ‘0’ (no confidence) to ‘100’ (complete confidence). Additionally, the subjects reported the number of falls in the last year. The Oxford scale was used to test for muscle strength (scale of ‘0’—no strength, to ‘5’—full range actively against resistance) and leg-length discrepancy (measured in centimeters). Finally, the subjects were tested at a gait laboratory, where four passive reflective markers were attached to the calcaneal tuberosity and the distal end of the second metatarsal, bilaterally. The subjects walked several times at a self-selected comfortable speed using their own footwear and devices on a 10 m long paved path, while 10 motion capture cameras (Qualisys, Gothenburg, Sweden) tracked the coordinates of the markers at a frequency of 120 Hz. A ceiling-mounted safety harness was applied for subjects with balance deficiencies. The subjects also performed the Timed Up-and-Go (TUG) test with the aforementioned markers still attached to their feet.

### 2.3. Data Analysis

The 3D coordinates of the four markers and the timings of initial contact and toe off were exported to a custom-made code (LabView 18, National Instruments, Austin, TX, USA), used to calculate all spatio-temporal parameters. Additionally, our code calculated the Coefficient of Variation (CV) for each spatio-temporal parameter, by dividing its Standard Deviation (SD) with its average value [28]. Additionally, the symmetry index between both legs was calculated by dividing the difference by the average values of the left and right legs [29].

### 2.4. Statistical Analysis

Statistical analyses were performed using Statistical Package for the Social Sciences (SPSS 27.0, Chicago, IL, USA). The Mann-Whitney test was used to describe differences between PPS subjects who use or did not use orthoses and/or assistive devices. Effect size estimates, r, for Mann–Whitney non-parametric tests were calculated according to [30]. Spearman’s rank test was used to test for correlations. Step-wise linear regression was used to find which gait parameters explained the risk and fear of falls. *p* < 0.05 was considered statistically significant.

## 3. Results

All of the data presented herein are available as Supplementary Materials to this publication.

### 3.1. Descriptive Statistics

Several gait measures differed between the subjects who did not require an orthotic and/or assistive device (n = 13) compared to the subjects who did require an orthotic and/or assistive device (n = 37). The statistically significant findings between the two groups are depicted in Table 2. There were no differences in the number of falls reported in the last year and other demographic or spatio-temporal gait parameters.

Twenty-three subjects (46%) reported no falls in the last year. Thirteen subjects (26%) had ABC score below 50%, which denotes low balance confidence associated with low level of physical functioning [31], 15 (30%) had ABC score between 50–80%, which denotes moderate balance confidence associated with moderate level of physical functioning, and 22 (44%) had ABC score above 80%, which denotes high balance confidence associated with a high level of physical functioning.

### 3.2. Correlations between Gait Parameters and Fall-Related Measures

The correlations between the falls parameters (ABC score and reported number of falls in the last year) and the gait and TUG measures are presented in Table 3. Only statistically significant findings are presented.

### 3.3. Regression Analyses

Three gait parameters explained 67.4% of the variance of the report of falls in the last year (*p* < 0.001): CV of the stance duration of the weak limb, CV of double support of the contralateral limb, and double support time (percent of the gait cycle) of the weak limb. The fitted regression model was number of falls = −2.198 + 0.694 (CV stance duration of the weak limb) − 0.213 (CV of the double support of the contralateral limb) + 0.227 (percentage of the double support of the weak limb).

The best predictor for the ABC score was the gait velocity, which explained 29.4% of its variance (*p* < 0.001). The fitted regression model was ABC score = 21.2 + 81.4 (gait velocity).

## 4. Discussion

The novelty of the current study is in its main finding, showing that the risk of falls in PPS patients can be predicted by simple parameters of the gait pattern: CV of the stance duration of the weak, CV of double support of the contralateral limb, and double support time (percent of the gait cycle) of the weak limb. These measures can be obtained in a clinical setting, but also using wearable sensors outside of the clinic. We believe that monitoring the gait pattern during daily activities will increase our ability to predict falls. Importantly, we believe our results may be proven a crucial factor in devising a real-time feedback system for the patient, alerting him or her regarding the need for rest or use of an assistive device, in order to prevent an imminent fall and possible related injury.

The gait characteristics of our study population are consistent with the results of our previous exploration of PPS gait, where we reported no association between the gait pattern of the PPS patients and sex, age, body mass index (BMI), education, and marital status [16]. Therefore, the gait pattern may essentially result from the physical status and compensation mechanisms. We also showed that PPS patients who use orthoses and/or walking aids ambulated with a smaller base width and better stance and swing durations symmetry compared to PPS patients who require these aids [16]. In the previous study, we did not calculate the CV of the spatio-temporal gait parameters. In the current study, we found that the gait variability differs between these two groups. Mainly, the CV of the base width, calculated separately for each leg, was smaller for the PPS group who use orthoses and/or walking aids compared to the PPS group who do not use them. This finding can be explained by the additional measurements described in Table 2. Specifically, the PPS group who require walking aids showed weakness of the quadricep muscle, which explains their lower score of balance confidence, i.e., lower ABC scores. Both muscle weakness and lower scores of balance confidence are most likely the cause for their wider base width, slower gait velocity and reduced cadence, also demonstrated by lower TUG scores. We surmise that this gait strategy of slower gait and wider base of support allows these patients to better control their gait variability, expressed by lower CV of the base width and double support duration.

The ABC scale is a valid and reliable tool for prediction of falls. It was shown to be a strong predictor falls for elderly people [32,33], individuals with Parkinson’s disease [34], post stroke patients [35], and individuals with multiple sclerosis [36]. In PPS patients, it was reported that participants (n = 415) who had higher ABC scores were less likely to have risk of falls (*p*  =  0.028) [37]. We therefore chose it as a secondary outcome measure for risk of falls. In our study, the ABC scores showed strong negative correlation with the TUG score and were moderately associated with the cadence and velocity of the gait. Additionally, the gait velocity was the best predictor of the ABC scale. It is not surprising that the gait velocity and cadence are related to the balance confidence. The patient will reduce gait velocity and cadence when feeling unsure of his or her stability while ambulating on different terrains. However, we expected that the time to complete the TUG, which includes both walking and turning, will also be compromised by low security in one’s balance. Although negative correlation between the ABC scale and the TUG score was reported in individuals with Parkinson’s disease (r = −372, *p* < 0.01) [38] and in older women (r = −0.39, *p* < 0.001) [39], proposed explanations for this unexpected finding were not presented by the authors. Since both the ABC scale and the TUG score were shown to be predictors of falls in various populations, we assume that there are different factors not shared between the two tests which serve as predictors of falls. These factors should be identified in future studies in order to better understand the motor and psychological mechanisms that contribute to the risk of falls.

The gait cadence and velocity were also negatively associated with the number of falls in the last year. PPS patients who walked slower and reported lower confidence in their balance also reported frequent falls. Here, we found positive correlation with the TUG score so that patients who took longer time to complete the TUG, also reported more falls. So much so that it seems that the motor components that play a role in the TUG are related to the actual risk of falls in PPS patients. Two more gait parameters positively associated with the frequency of falls were the symmetry index of the swing duration and the CV of the step length of the weak limb. That between-limb asymmetry of the swing duration and high variability of the step length in the weak limb were associated with a higher number of falls. These two parameters are related to one another, since asymmetry in the swing durations between the limbs means that the patient may drop down his or her leg sooner (or later) during the gait cycle, thereby decreasing (or increasing) the step length. This might cause an inadequate response to the natural movement of the body’s center of gravity during locomotion, resulting in loss of balance and inevitable fall. However, these two gait parameters were not the best predictors for the reported occurrence of falls. The three predictors of falls were the CV of the stance duration of the weak limb, CV of double support of the contralateral limb, and double support time (percent of the gait cycle) of the weak limb. Double support duration is an important gait measure found to increase with age as an attempt to increase stability [40]. Additionally, the double support duration was found to be approximately 2% (of the gait cycle) higher in fallers compared to non-fallers [41]. However, to the best of our knowledge, this is the first study to use the CV of the double support duration, along with other spatio-temporal gait parameters, in order to predict frequency of falls. Indeed, the CV of the double support duration was another predictor of falls in PPS patients, along with the CV of the stance duration. These findings might prove to be important when devising a predictive algorithm to monitor and provide real-time notifications to patients at risk of falls using the state-of-the-art technology.

Our results are supportive of new algorithms and applications that allow continuous measurement of gait velocity and cadence via the smartphone, placed on the body, in a bag, or on a belt [42,43]. Additional technologies that acquire spatio-temporal gait parameters include E-Textile-Based wearable socks [44], Inertial Measurement Units (IMU) [45], Hall-effect sensors [46], and smartwatches [47]. A thorough list of wearable sensors used for gait analysis is detailed in [46]. These technologies allow remote patient monitoring. Importantly, we believe that our results support the integration of real-time feedback and alerting algorithms that can protect the patient in case of dangerous gait patterns that predict an imminent fall.

The limitations of this study include the subjective report of the subjects regarding their history of falls in the last year and balance confidence. Although these reports might be sensitive to the subject’s interpretation, they are frequently used for the study of risk of falls [1,11,48]. Another limitation is the single-day measurements in lab settings that might not be demonstrative of the daily gait pattern of the patient.

## 5. Conclusions

We conclude that the gait variability, easily measured by wearables or pressure-sensing mats, is an important predictor of falls in PPS population. Therefore, gait variability might be an efficient tool before devising a patient-specific fall prevention program for PPS patients.

## 6. Future Research

It would be interesting to collect gait data over a period of time from individuals with PPS walking at their natural environment. For example, 3 IMUs placed on the pelvis and ankle, could supply CV data of temporal parameters, while simultaneously recording occurrences of falls. Consequently, the data regarding frequency of falls will not depend on the memory or interpretation of the patient and interesting data of walking on various terrains—e.g., stairs and slopes—might add to our knowledge about the gait characteristics and fall risk in individuals with PPS.

## Figures and Tables

**Table 1:** Literature review of studies that performed instrumented gait analysis in post-polio syndrome (PPS) patients. single subject case-studies were not included in the review.

Primary Outcome Measures of Gait	Technology	N	Main Finding	Fall-Related Data	Reference
Forces and moments passing through knee-ankle-foot orthoses	Load cell in the orthosis	4	Knee joint forces and moments) were composed of knee flexion moments and axial compression forces	None	[17]
Knee extension moments while walking with an orthosis	Load transducer, motion capture system and force plates	4	Adding a dorsiflexion stop at the orthotic ankle decreased knee flexion moments	None	[18]
Gait symmetry while using difference orthoses	Motion capture system	7	Powered orthosis affected gait symmetry in the base of support, swing time, stance phase percentage, and knee flexion during swing phase	None	[19]
Kinematics and spatiotemporal parameters while walking with different orthoses	Motion capture system	7	The powered knee-ankle-foot orthosis reduced gait speed and step length and increased stance phase percentage, knee flexion, and hip hiking	None	[20]
Kinematics and energy consumption while walking with an electromechanical orthosis	Motion capture system and force plates	4	Increased knee flexion in the swing phase with the orthosis	None	[21]
Effect of an orthosis with drop lock vs. powered knee joints on gait speed and distance	Stop watch and distance measurement	7	Walking with the powered orthosis reduced walking speed and distance	None	[22]
Timed Up-and-Go test, the Comfortable and the Fast Gait Speed tests, and 6-Minute Walk test	Stop watch	30	Test-retest reliability was established	None	[23]
Kinematics and 6-Minute Walk test	Motion capture system	18	Walking speed was negatively correlated with the increased hip flexion	None	[24]
Timed Up-and-Go test and 6-Minute Walk test	Stop watch	81	Higher score in the 6-min walk test reduced the risk of fall	Fall history and fear of falling	[11]
Kinematics and kinetics while walking with dorsiflexion-restricting ankle-foot orthoses	Motion capture system and force plates	16	The orthosis increased gait speed and forward progression of the center of pressure in mid-stance. It reduced ankle dorsiflexion and knee flexion in mid- and terminal stance	Fear of falling	[25]
Spatio-temporal parameters and symmetry indices	Motion capture system	26	Subjects who did not report falling indoors in the last 6 months had higher gait velocity and cadence, shorter double support stance and step durations, longer step length and better step length symmetry index.	Number of falls	[16]
Ten-meter walk test and number of steps per day	Pedometer	128	Subjects with one or more falls in the preceding year had slower gait speed and higher fear of falling	Fall history and fear of falling	[26]

**Table 2:** Significant differences found in all study measures between subjects who did not require an orthotic and/or assistive device compared to the subjects who required an orthotic and/or assistive device. Measures include: The Activities-specific Balance Confidence Scale = ABC score, and the Timed up and Go (TUG). CV = Coefficient of Variability.

Measure	Without Walking Aids (n = 13)	With Walking Aids (n = 37)	*p*	r
Quadriceps muscle strength (0–5)	3.2 ± 1.3	1.3 ± 1.2	0.006	−0.445
ABC score (0–100)	94.5 ± 37.2	64.7 ± 34.5	0.013	−0.352
TUG (s)	17.8 ± 7.4	26.4 ± 12.9	0.007	−0.392
Gait velocity (m/s)	0.82 ± 0.22	0.56 ± 0.22	0.001	−0.461
Cadence (steps/min)	89.2 ± 14.1	74.4 ± 15.5	0.007	−0.383
Base width of the weak limb (cm)	8.9 ± 4.8	15.1 ± 6.8	0.009	−0.396
Base width of the contralateral limb (cm)	10.9 ± 5.0	15.9 ± 7.1	0.036	−0.316
CV double support of the weak limb	47.5 ± 41.2	18.4 ± 27.4	0.027	−0.334
CV base width of the weak limb	45.9 ± 34.1	22.7 ± 13.3	0.017	−0.359
CV base width of the contralateral limb	44.2 ± 27.6	28.2 ± 21.5	0.030	−0.328

**Table 3:** Correlations between the fall parameters (activity-specific Balance Confidence Scale = ABC score and number of falls in the last year) and the gait and Timed Up-and-Go (TUG) measures. CV = Coefficient of Variability.

Measure	ABC Score	Falls in the Last Year
TUG (s)	−0.604, <0.001	0.491, 0.001
Gait velocity (m/s)	0.552, <0.001	−0.400, 0.007
Gait cadence (steps/min)	0.483, <0.001	−0.405, 0.006
Swing duration symmetry index	-	0.433, 0.003
CV Step length of the weak limb	-	0.442, 0.004

## Data Availability

Data is contained within the article or Supplementary Materials.

## Supplementary Materials

The following are available online at https://www.mdpi.com/article/10.3390/biology10111110/s1. SPSS file.
